# Assessment of the inclusion of vaccination as an intervention to reduce antimicrobial resistance in AMR national action plans: a global review

**DOI:** 10.1186/s12992-022-00878-6

**Published:** 2022-10-17

**Authors:** Lotte van Heuvel, Saverio Caini, Michel L. A. Dückers, John Paget

**Affiliations:** 1 Nivel, Netherlands Institute for Health Services Research, Otterstraat 118, 3513 CR Utrecht, The Netherlands; 2 ARQ National Psychotrauma Centre, Diemen, the Netherlands; 3 Faculty of Social and Behavioural Sciences, University of Groningen, Groningen, the Netherlands

**Keywords:** National Action Plans, Global action plan, AMR, Vaccination, Policy

## Abstract

**Background:**

Vaccination can reduce antibiotic use by decreasing bacterial and viral infections and vaccines are highlighted in the WHO Global Action Plan on Antimicrobial Resistance (AMR) as an infection prevention measure to reduce AMR. Our study aimed to analyze whether WHO Member States have developed AMR national action plans that are aligned with the Global Action Plan regarding objectives on vaccination.

**Methods:**

We reviewed 77 out of 90 AMR national action plans available in the WHO library that were written after publication of the Global Action Plan in 2015. Each plan was analyzed using content analysis, with a focus on vaccination and key components as defined by WHO (I. Strategic plan (e.g. goals and objectives), II. Operational plan, III. Monitoring and Evaluation plan).

**Results:**

Vaccination was included in 67 of 77 AMR plans (87%) across all WHO Regions (Africa: *n* = 13/13, the Eastern Mediterranean: *n* = 15/16, Europe: *n* = 10/14, the Americas: *n* = 8/8, South-East Asia: *n* = 8/11, and the Western Pacific: *n* = 13/15). Pneumococcal and influenza vaccination were most frequently highlighted (*n* = 12 and *n* = 11). We found indications that vaccination objectives are more often included in AMR plans from higher income countries, while lower income countries more often include specific vaccines. The key WHO components of national action plans were frequently not covered (I. 47% included, II. 57%, III. 40%). In total, 33 countries (43%) included indicators (e.g. strategic objectives) to capture the role of vaccines against AMR.

**Conclusions:**

While vaccination to reduce AMR is seen as an important global public health issue by WHO, there appears to be a gap in its adoption in national AMR plans. Country income levels seem to influence the progress, implementation and focus of national action plans, guided by a lack of funding and prioritization in developing countries. To better align the global response to AMR, our review suggests there is a need to update national action plans to include objectives on vaccination with more focus on specific vaccines that impact antibiotic use.

**Supplementary Information:**

The online version contains supplementary material available at 10.1186/s12992-022-00878-6.

## Background

Antimicrobial resistance (AMR) remains an increasing threat to global health and it has been projected that in 2050, 10 million deaths a year will be attributable to drug-resistant bacteria [1, 2]. Countries worldwide have joined forces to fight AMR, for example through global one health partnerships, antibiotic stewardship policies, and global and national action plans. Member States of the World Health Organization (WHO) have developed AMR national action plans after their endorsement of the WHO Global Action Plan on AMR (GAP-AMR) in 2015. These plans include strategic objectives to tackle AMR which focus on awareness, surveillance, infection prevention and control (IPC), antimicrobial use, economic investment in new medicines, and other country-specific objectives [2]. In recent years, however, vaccination has been increasingly seen as another effective approach to reduce AMR [3–6].

The WHO (2015) was one of the first global actors to acknowledge that “*vaccination, where appropriate as an infection prevention measure, should be encouraged.*” Vaccination is highlighted in Objective 3 of the WHO GAP-AMR [2]: ‘Reduce the incidence of infection through effective sanitation, hygiene and infection prevention measures’, with vaccination reducing AMR by: (1) preventing infectious diseases whose treatment would require antimicrobial medicines, (2) reducing the prevalence of viral infections which can give rise to secondary infections that require antibiotic treatment, and (3) preventing diseases that are (becoming) untreatable owing to AMR. Vaccination can also reduce antibiotic use by decreasing transmission of infections in the community through herd immunity [3].

More recently, WHO (2020) has published an Action Framework ‘Leveraging Vaccines to Reduce Antibiotic Use and Prevent AMR’ in which they state that the increased uptake of Influenza, Pneumococcal vaccines (PCV), Typhoid vaccines (TCV) and *Haemophilus Influenzae* type B (Hib) vaccines should be prioritized for its impact on antibiotic use and AMR. Solid evidence suggests that influenza and pneumococcal vaccination can reduce antibiotic use in different risk groups (e.g. young children, older adults and people with chronic medical conditions) [7, 8] and that vaccination could help prevent secondary bacterial infections which often require antibiotic treatment, such as *Streptococcus pneumoniae* [3, 9, 10]. *S. pneumoniae* was responsible for almost 600.000 deaths in 2019 and one in five deaths attributable to bacterial AMR occurred in children < 5 years [11]. Research shows that the introduction of PCV has decreased the rate of drug-resistant invasive pneumococcal disease [10, 12] and averted considerable antibiotic treatment failures and AMR-related deaths [13]. It was also noted after the introduction of the Hib conjugate vaccines that there was not only a decrease of Hib disease rates in Canada [14], but also a 10.2% decline in the prevalence of resistant strains (e.g. β-lactamase) in over a decade in the United States [15]. These findings have led to the promotion of including vaccination in AMR policies and national action plans by the scientific community and international health organizations such as WHO.

To our knowledge [16], it is unclear if the WHO Member States are aware of the added benefits of vaccination in the fight against AMR and if they address vaccination in their AMR national action plans. National action plans should be developed and implemented by the Member States to respond to the growing threat of AMR and, according to the 2015 World Health Assembly resolution [17], “all Member States are urged to have in place, within two years of the endorsement of the draft action plan by the Health Assembly, national action plans on antimicrobial resistance that are aligned with the global action plan and with standards and guidelines established by intergovernmental bodies.” As of October 2021, 148 out of 194 countries have finalized their national action plans aligned with objectives of the GAP-AMR [18]. These national action plans should therefore include vaccination as an infection prevention measure and focus on expanding the use of licensed vaccines to control AMR [3, 19].

Our study aims to analyze whether AMR national action plans are aligned with the Global Action Plan on AMR and include strategic objectives on vaccination. It presents an overview of vaccination in national action plans on AMR, focusing on PCV, TCV, Hib vaccines, influenza vaccines, rotavirus and measles vaccines.

## Methods

**Fig. 1:**
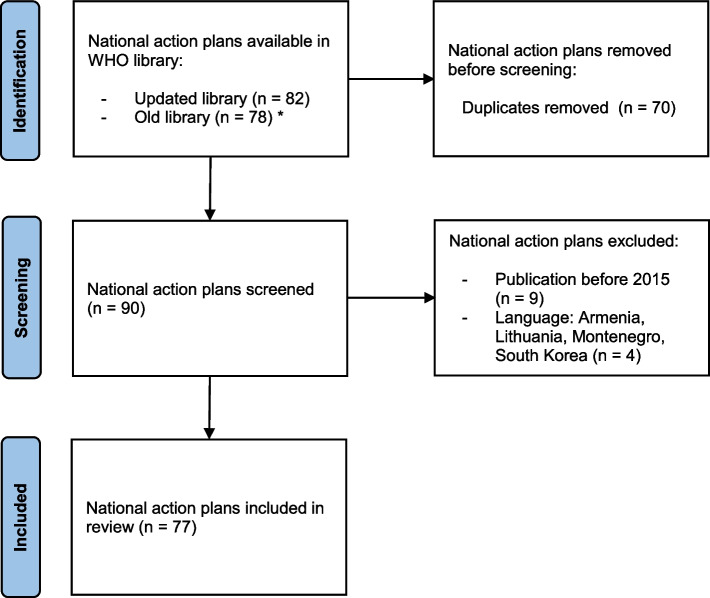
Selection of national action plans on AMR. *On 16 June 2021, we noticed that the WHO library of AMR national action plans was updated and the old library (webpage) was not accessible. At that moment, not all plans were reported in the updated library and therefore we included plans from both libraries. The final search was done on 22 July 2021

We included national action plans on AMR that were available online on 22 July 2021 in the WHO library of AMR national action plans [20], see Fig. 1. Plans were screened based on publication date and language. Only national plans written after publication of the AMR Global Action Plan in 2015 were included to analyze alignment with the strategic objectives on vaccination. We analyzed national action plans available in the following languages: English, French, Spanish, Portuguese, Italian and German. Each plan was downloaded from the WHO library website [20]. We included AMR plans from all WHO regions, including the African Region (AFR), Eastern Mediterranean Region (EMR), European Region (EUR), Region of the Americas (RAM), South-East Asia Region (SEAR), and the Western Pacific Region (WPR). In addition, countries with different income levels were selected, as based on the World Bank country classification [21]: Low-Income Countries (LIC), Lower-Middle-Income Countries (LMIC), Upper-Middle-Income Countries (UMIC), High-Income Countries (HIC).

The AMR national action plans were reviewed based on the information on vaccination and the operational structure. The 2016 manual for developing AMR national action plans [22] was used to select key components of action plans, as defined by the World Health Organization, Food and Agriculture Organization and the World Organisation for Animal Health, including: I. a strategic plan (e.g. goals and objectives), II. an operational plan (including detailed budgeting and funding), and III. a monitoring and evaluation (M&E) plan (see Table 1). This review focused on six licensed vaccines that are recommended by WHO [3] to use for its potential impact on AMR: PCV, TCV, Hib vaccines, influenza vaccines, rotavirus vaccines and measles-containing vaccines.

**Table 1:** Key components of national action plans as defined in the 2016 WHO manual for developing AMR national action plans [22]

	WHO key components of national action plans	
I	Strategic plan	- Goals and objectives to control AMR - Priority areas and interventions
II	Operational plan	- Activities and sub-activities, implementation arrangements, timetable, responsible entities - Detailed budgeting and costing, source of funding (for each activity)
III	Monitoring and Evaluation plan	- Performance indicators of achievement (for the objectives and activities) - Targets and timelines - Data collection and reporting methods

Information was extracted from the national action plans by three researchers (LvH, SC and JP). Each plan was analyzed using content analysis based on two main factors: (1) information on vaccination, with a focus on promoting vaccination, optimizing vaccination coverage and/or strengthening vaccination programs targeting the six selected vaccines, and (2) the WHO key components of national action plans. The analysis also focused on similarities with the GAP-AMR (as defined by Munkholm and Rubin [23]), extensiveness and explanations of concepts and objectives, involvement of experts in the development of the action plans, and the inclusion of evidence-based statements. All relevant information was extracted and the variables were categorized to compare the national action plans by income level and WHO region (see Supplementary File 1).

The association between income and vaccination (focus on objectives related to vaccination and the inclusion of specific vaccines) was explored using multi-level logistic regression analysis, including WHO region as a random effect (countries are nested in six regions) and income level as a fixed effect. The significance threshold was set at .05.

## Results

This study included 77 national action plans on AMR from the WHO Member States, available in the WHO library of AMR national action plans. This selection included countries from every WHO region: AFR (*n* = 13), EMR (*n* = 16), EUR (*n* = 14), RAM (*n* = 8), SEAR (*n* = 11), and WPR (*n* = 15). The income level of these countries covered: LIC (*n* = 9), LMIC (*n* = 24), UMIC (*n* = 20), and HIC (*n* = 24). Most national action plans were published in 2017 (*n* = 32), following 2018 (*n* = 15) and 2019 (*n* = 15). The Supplementary File 1 provides an overview of all national action plans included in this study.

### Information on vaccination

**Fig. 2:**
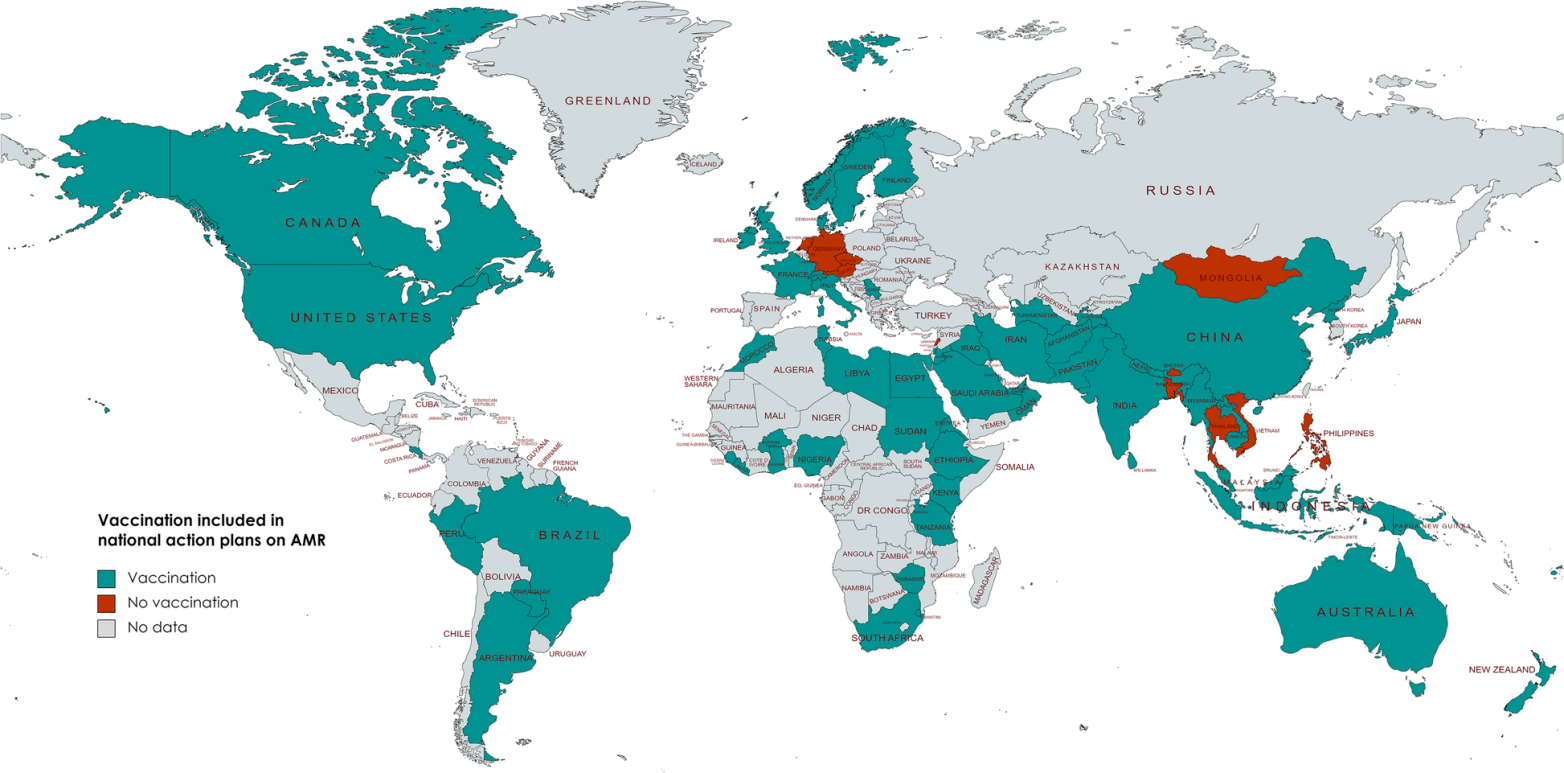
World map of AMR national action plans including vaccination

Vaccination was mentioned in 67 out of 77 national action plans (87%) across all WHO regions, see Fig. 2. Ten countries, in WHO EUR (*n* = 4), SEAR (*n* = 3), WPR (*n* = 2), and EMR (*n* = 1), did not mention (human) vaccination at all in their national action plans, while all countries in the WHO African Region (*n* = 13) and American Region (*n* = 8) have included vaccination. Seventeen countries reported information on the effect of specific vaccines on AMR, with 10 national action plans covering multiple vaccines as shown in Table 2. The mean number of vaccines described in the 17 AMR plans is 2.18 (95% CI 1.59 – 2.76; Med = 2; SD = 1.13). All LIC countries (*n* = 9) include vaccination, however only one LIC country (Afghanistan) also mentions specific vaccines in its national action plan. One LIC (11%), five LMIC (21%), two UMIC (10%), and nine HIC (38%) include specific vaccines.

**Table 2:** Overview of 17 out of 77 national action plans that include specific vaccines

	National action plans	PCV	Influenza	Hib	TCV	Measles	Rotavirus
1	Afghanistan	x	x	x	x		
2	Barbados		x			x	
3	Finland	x	x		x		
4	India	x	x	x	x		
5	Iran	x	x	x			
6	Italy	x	x			x	
7	Japan	x	x	x			
8	Malaysia	x			x	x	
9	Morocco	x		x			x
10	Myanmar		x				
11	Norway		x				
12	Switzerland	x					
13	Tunisia	x	x				
14	United Arab Emirates		x				
15	United Kingdom	x					
16	United States	x					
17	Zimbabwe						x
	**Total**	**12**	**11**	**5**	**4**	**3**	**2**

Of the 67 national action plans that highlighted vaccination, 33 countries (49%) developed specific indicators to promote vaccination (and specify the value of vaccination on AMR prevention and control) while 34 countries (51%) merely mentioned vaccination as an example. Table 3 shows this data specified for each vaccine.

**Table 3:** Total of 17 national action plans discussing vaccination, either as indicator or example

Vaccination included	As indicator	As example
PCV	6	6
Influenza vaccine	7	4
Hib vaccine	4	1
TCV	3	1
Measles vaccine	1	2
Rotavirus vaccine	0	2

Note: vaccination is either included in national action plans as indicator (e.g. objective, strategy, goal, action, intervention, priority area, measure or key activity) or as example of a type of vaccination that can impact AMR

### Country comparison

We noticed that AMR national action plans overlap (e.g. textual overlap) with other plans across WHO regions and income groups, focusing on objectives on vaccination and specific vaccines included in the plans (e.g. Objective 4.6.1.3 of the Afghanistan and Indian national action plan is identical). Countries include more objectives on vaccination (43%) compared to specific vaccines (22%). Table 4 presents these results for each WHO region.

**Table 4:** Vaccination (objectives and specific vaccines) included in 77 AMR national action plans by WHO region

WHO region	Objective on vaccination	Specific vaccines
EMR	6 (38%)	5 (31%)
WPR	7 (47%)	2 (13%)
SEAR	4 (36%)	2 (18%)
RAM	3 (38%)	1 (14%)
EUR	6 (43%)	2 (18%)
AFR	7 (54%)	5 (29%)
**Total**	**33**	**17**

**Fig. 3:**
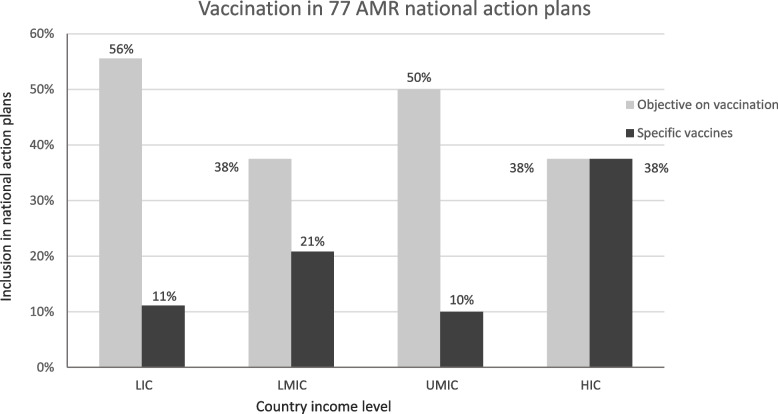
Vaccination (objectives and specific vaccines) included in 77 AMR national action plans by income level

Figure 3 shows the inclusion of objectives on vaccination or specific vaccines (e.g. PCV, influenza vaccine, Hib vaccine, TCV, measles and/or rotavirus vaccination) in 77 national action plans, specified by country income level. Details on the 33 countries that include objectives on vaccination can be found in Supplementary File 1.

We assessed the association between income and vaccination using multilevel logistic regression and found that an increase of income is accompanied by a lower probability of including specific vaccines in AMR plans (OR = .77; *p* = .18; 95% CI .52-1.13). We found a weaker association, non-significant as well, in the opposite direction between income and the probability of including vaccination objectives in action plans; here an increase in income is accompanied by a slightly higher probability (OR = 1.05; *p* = .74; 95% CI .78-1.43). The multilevel model shows that regional variation is larger when it comes to the inclusion of specific vaccines in national action plans compared to objectives on vaccination (see Supplementary File 2).

### Key components of national action plans

For this analysis we excluded 7 plans published in 2015, before publication of the 2016 WHO manual for developing national action plans. Out of the remaining 70 action plans, 21 (30%) included no key components and 19 (27%) included all key components (with 10 out of 19 countries in WHO EMR). Most countries include an operational plan (57%), followed by a strategic plan (47%) and M&E plan (40%). Table 5 shows the total number of plans that include a key component (i.e. component is specifically mentioned in the text) per income level and WHO region, although these key components are often not related to promoting or strengthening vaccination. In total, 11 strategic plans, 11 operational plans and 7 M&E plans include a specific objective or activity on vaccination. Only five AMR plans (7%) included all key components, including cost and funding, with a focus on vaccination.

**Table 5:** Key components included in 70 national action plans published after 2016

	Strategic plan	Operational plan	M&E plan
**Income level**			
LIC	6 (67%)	8 (89%)	6 (67%)
LMIC	12 (50%)	17 (71%)	12 (50%)
UMIC	10 (50%)	10 (50%)	7 (35%)
HIC	5 (21%)	5 (21%)	3 (13%)
**WHO region**			
EMR	11 (69%)	14 (88%)	13 (81%)
WPR	2 (13%)	7 (47%)	2 (13%)
SEAR	5 (45%)	2 (18%)	2 (18%)
RAM	4 (50%)	3 (38%)	1 (13%)
EUR	2 (14%)	3 (21%)	1 (7%)
AFR	9 (69%)	11 (85%)	9 (69%)
**Total**	**33**	**40**	**28**

#### Strategic plan

The strategic objectives in national action plans are not always specified in the strategic plan, as less than half of the 70 AMR plans (*n* = 33; 47%) included a strategic plan. The other 37 plans presented the objectives or strategies to tackle AMR throughout the text, focusing mainly on awareness, surveillance, infection prevention and control, antibiotic stewardship, and research and innovation. These main objectives are similar to the Global Action Plan on AMR, with additional country-specific objectives.

As based on the Global Action Plan, the following objectives were recurring in the AMR national action plans: (1) increase national awareness and understanding of AMR, (2) enhance national surveillance of AMR, (3) reduce the incidence of infections and contain the spread of antimicrobial-resistant organisms through effective IPC (e.g. sanitation and hygiene), (4) optimize the appropriate use of antimicrobials in humans, animals and agriculture, and (5) increase investment in research and development (R&D) for new antibiotics, vaccines, tools and other interventions.

Vaccination was often referred to in objectives related to infection prevention and control (strengthen, improve and promote vaccination programs: 67%) and research and innovation (prepare economic arguments for sustainable investment in new drugs, diagnostics and vaccines: 52%). Out of the 33 strategic plans, 11 plans (33%) included the promotion of vaccination or strengthening of vaccination programs as a strategic objective.

#### Operational plan

An implementation and operational plan was included in 40 out of 70 national action plans (57%). For each activity and sub-activity (what), these plans include a responsible entity (who), a timeline (when), and indicators, milestones or targets (how). Most operational plans are included in WHO EMR and AFR (see Table 5). Promoting or strengthening vaccination is a specific activity included in the implementation and operational plan of 11 national action plans (16%).

##### Costs and funding

Of the 70 national action plans published after 2016, costs and funding were described in 23 plans (33%). The costs or estimated budget often, but not always, included a source of funding and are described for each objective and activity. Costs and/or funding is described for five objectives and activities specifically related to promoting vaccination, included in the national action plans of Eritrea, Libya, Nigeria, Tanzania and Tunisia (WHO EMR and AFR).

#### Monitoring and evaluation plan

Twenty-eight countries (40%) included a M&E plan. For each activity and sub-activity, the plan describes the indicator, method and targets (by year). Only seven national action plans (10%) described monitoring and evaluation of objectives related to promoting or strengthening vaccination coverage: Eritrea, Nigeria, Pakistan, Sierra Leone, Tanzania, Tunisia and Zimbabwe (WHO EMR and AFR).

## Discussion

Globally, in a total of 77 AMR national action plans, vaccination is frequently mentioned (87%) but is not always recognized as a main objective in these plans (43%). Only five countries (6%) included all of the WHO key components (with a focus on promoting or strengthening vaccination) in their national action plan; these include, a strategic plan, an operational plan with costs and funding, and a monitoring and evaluation plan. We identified a clear variation in themes and objectives discussed in national AMR plans. At this moment, awareness to the potential of vaccination to reduce antibiotic use is low and a confirmed effective strategy is not utilized to its full potential in all WHO Member States. When updating AMR national action plans, countries should include strategic objectives to increase and promote vaccination and focus on vaccination programs that target specific vaccines.

This review shows that vaccination is not highlighted in every AMR action plan, while there is considerable evidence on the effect of vaccination on antibiotic use (and AMR). Two systematic reviews by Buckley [8] and Doherty [7], including RCTs from WHO EUR and RAM, have shown the effect of pneumococcal and influenza vaccination on reducing antibiotic usage among all age groups. These two vaccines were also highlighted most often in the national action plans included in our study (pneumococcal: 16%; influenza: 14%), in low- to high-income countries mainly in WHO EUR and EMR. Interestingly, multiple countries (e.g. Jordan, Kenya, Mauritius, Nepal, Pakistan and the Philippines) have mentioned outbreaks of measles or typhoid fever in their national action plan, but vaccination is not mentioned as a possible solution. Nonetheless, international experts have acknowledged the role of vaccination in AMR. In a survey of Gavi (the Vaccine Alliance), experts attributed the highest value to pneumococcal, typhoid and malaria vaccines in relation to AMR [24]. Italian vaccine experts too have recognized the role of existing vaccines in limiting AMR, in particular pertussis, meningococcus, measles and varicella, and they agreed that the role of vaccination against AMR should be expressed in Italian national vaccination guidelines [25]. Vaccination is therefore seen as an effective approach to reduce AMR by many international experts and it is highlighted in literature, but this review shows its potential is not fully realized by countries in policy and action plans.

A lack of implementation of available action plans is one of the main issues with realizing effective vaccination strategies against AMR. Two main barriers for implementation are a lack of funding and a lack of (political) prioritization of AMR, especially in LMIC [6]. Global progress and the implementation of AMR national action plans is measured by country self-assessment according to the WHO tripartite self-assessment survey (TrACSS) [26]. Over the years, these surveys have shown that the number of countries with developed action plans has increased, however many of these plans do not include an operational or monitoring plan with funding sources. For example, only 19.9% of countries worldwide reported having government funded AMR campaigns targeting key stakeholders in 2019-2020 [26]. In our study we found that 33% out of 70 national action plans published after 2015 included detailed budgeting and funding. The difficulty of implementing AMR national action plans, and a lack of budget or financial resources, is recognized by international public health experts [16]. AMR stakeholders are concerned that the priority of AMR on the political agenda declines as it competes with other, more immediate, public health topics [6]. To strengthen the implementation of AMR national action plans, Anderson and Mossiales (2020) have developed a framework to improve governance [27]. The governance framework focuses on policy design, implementation tools, and monitoring and evaluation to improve the quality of governance in these areas. These governmental strategies could help to build better engagement among stakeholders and coordinate actions that facilitate quality improvement.

At the moment, the actions and objectives included in AMR national action plans differ between countries, while WHO directs all plans to be aligned with global frameworks like the GAP-AMR and to focus on five strategic AMR objectives. Research by Munkholm and Rubin (2020) shows that there is strong alignment with the objectives and corresponding actions outlined in the Global Action Plan, but not in the actual implemented policies [23]. Experts globally have expressed concerns about countries that apply ‘copy-and-paste’ exercises from the GAP-AMR WHO template without adapting to country specifics [6]. Poorer WHO Member States are more likely to align with the GAP-AMR: developing (often African) countries display high values linked to verbatim overlap compared to developed (often European) countries [23]. The WHO TrACSS also shows that levels of achievement towards objectives of the GAP-AMR (e.g. surveillance, training and education on AMR, national IPC programs) significantly differ based on income group, with higher levels of achievement in HIC, highlighting the need for technical and financial support for national action plan implementation in LIC [16]. Currently, LIC experience a lack of enabling environments with regards to funding, coordination and political leadership [6]. Similarly, our review found indications of a possible association between the income level and a focus on objectives on vaccination, with HICs more likely to include vaccination objectives in their action plans. These differences between income level and vaccination strategies requires further examination in relation to cultural, political and socioeconomic determinants.

The differences in the quality of the action plans and their implementation can lead to inequity between countries in the fight against AMR. The SARS-CoV-2 pandemic has reaffirmed the importance of vaccination against infectious diseases. While AMR is often referred to as a ‘silent pandemic’, it can affect as many people as, and be influenced by, the COVID-19 pandemic [28, 29]. Hence it is important to find new solutions in the fight against AMR, such as vaccination, while being aware of changing priorities and changes to the global health agenda due to COVID-19. One aspect to be focused on is how the development and adoption of national action plans can be increased in all WHO Member States. There are organizations (e.g. the Fleming Fund) which support countries by guiding their implementation with a comprehensive program that goes beyond the provision of templates and guidelines [6]. At this moment, a total of 113 WHO Member States have signed the WHO Call to Action on AMR 2021 [30], and pledged to make efforts to have a fully funded, implemented and evaluated multisectoral AMR national action plan. This review shows additional areas to improve the national action plans that can be used by countries when updating or developing their plans, aiming for more similarities between action plans and public health policies with equal efforts between countries in the fight against AMR.

One limitation of this study is the use of the WHO database to select AMR national action plans. The library collects existing, publicly available, officially approved plans, with 83 plans available on October 2021 [20]. Though as of October 2021, 148 out of 192 Member States have finalized their national action plan aligned with the objectives of the GAP-AMR and 38 countries are in the process of developing their action plan [18]. We cannot rule out whether our decision to only work with official WHO approved action plans might have led to selection bias impacting the validity of this study. Still, we included a large sample (*n* = 77) of AMR plans from countries with diverse income levels from different regions and we belief that this sample is representative of the income and regional diversity of all WHO Member States. The sample size does require considerations with the interpretation of (regional) country comparisons and the small effect size identified through our data analysis. It is possible that a larger sample could result in a significant association between income and specific vaccines, and it would be therefore valuable to perform a more detailed country comparison in the future also as the WHO library is continuously updated when new plans are available.

This study gives an overview of countries that express attention to vaccination in their AMR national action plan. Future studies should also review country specific vaccination programs (e.g. influenza vaccination programs) or AMR policies (e.g. hospital guidelines and antimicrobial stewardship policies) that relate to this topic. Differences in existing vaccination policies and vaccination coverage rates between WHO regions [31] might explain the cross-country contrast in the attention to expanding the use of vaccines to control AMR. Our study is limited to national action plans, but a report by van Heuvel et al. (2020) shows that a considerable number of reports by public health organizations also refer to the effect of vaccination on AMR, by preventing infections and reducing the need for and use of antibiotics [16]. The WHO has a key role to play in raising attention to vaccines against AMR and guiding countries in the development of their national action plans. Priority actions identified in the recent WHO Vaccination Action Framework are a good first step to maximize the impact of vaccines in tackling AMR [3]. One of these actions focuses on the consistent inclusion of vaccines as interventions planned for the use against AMR in AMR national action plans. As discussed, this review helps in identifying obstacles surrounding these interventions and identifies areas to revise and emphasize when developing and updating AMR plans.

In short, we recommend that the progress of AMR national action plans with regards to the inclusion of vaccination is reviewed every 5 years (i.e. when national action plans are updated) complementary to tracking national progress in the implementation of the plans, as it will take some time until the gap between objectives and practice is bridged. This means the implementation of AMR policies should not only be monitored by country self-assessment (e.g. TrACSS [26]), but also through independent research and organizations that have a multi-country perspective. Besides the periodic updates of national policies, it is also important to invest in further improvement of the GAP-AMR itself as a guidance framework to increase effective implementation in relation to country-specific circumstances.

## Conclusions

This review is a first step to study the inclusion of vaccination in (inter)national policies. Extensive research has shown that vaccination can reduce antibiotic use and AMR, hence there is a benefit to include vaccination in global AMR policies and national action plans. Vaccination is frequently discussed in national AMR plans, however this topic is often not included in strategic objectives or outlined in detail in implementation and evaluation plans. Countries have paid most attention to pneumococcal and influenza vaccination and the attention to these and other specific vaccines appears to be linked to cultural, political and socioeconomic country characteristics that warrant further inquiry. The present lack of detail and awareness regarding the implementation of the national AMR plans and interventions related to vaccination are points of concern and require new, effective governance strategies to increase the global attention to vaccination. Thus, to better align the global response to AMR and national activities, our review suggests there is a need to update national action plans on AMR to include objectives on vaccination and specify vaccines of interest for priority action.

## Supplementary Information

**Additional file 1.**

**Additional file 2.**

## Data Availability

All data generated or analyzed during this study are included in this published article [and its supplementary information files].
