# Correction to: Assessment of the inclusion of vaccination as an intervention to reduce antimicrobial resistance in AMR national action plans: a global review

**DOI:** 10.1186/s12992-023-00951-8

**Published:** 2023-08-08

**Authors:** Lotte van Heuvel, Saverio Caini, Michel L. A. Dückers, John Paget

**Affiliations:** 1 Nivel, Netherlands Institute for Health Services Research, Otterstraat 118, Utrecht, 3513 CR The Netherlands; 2 ARQ National Psychotrauma Centre, Diemen, the Netherlands; 3 Faculty of Social and Behavioural Sciences, University of Groningen, Groningen, the Netherlands

**Correction to: Globalization and Health (2022) 18:85**

10.1186/s12992-022-00878-6

Following publication of the original article [1], the authors identified errors in Table 4 and in the analysis provided in Additional File 2.

Concerning Table 4, some of the values provided in the third column had been aligned with the wrong WHO region. The alignment of the values has since been corrected in the published article.

**Table 4:** Vaccination (objectives and specific vaccines) included in 77 AMR national action plans by WHO region

WHO region	Objective on vaccination	Specific vaccines
EMR	6 (38%)	5 (31%)
WPR	7 (47%)	2 (13%)
SEAR	4 (36%)	2 (18%)
RAM	3 (38%)	2 (25%)
EUR	6 (43%)	5 (36%)
AFR	7 (54%)	1 (8%)
**Total**	**33**	**17**

Regarding Additional File 2, the logistic multi-level regression analyses results in this file were inconsistent with the results that are provided in Figure 3. This inconsistency was the result of an error in the statistical analyses performed by the authors, whereby the authors mistakenly presented the association between the income of countries and the likelihood of specific vaccines and vaccination objectives being included in AMR plans. Additional File 2 has since been corrected in the published article.

Per the correction of Additional File 2, the following corrections have been made (see the corrections highlighted in bold) in:

The ‘Country comparison’ subsection of the Results:

‘We assessed the association between income and vaccination using multilevel logistic regression and found that an increase of income is accompanied by a **higher** probability of including specific vaccines in AMR plans **(OR = 1.59; p = .11; 95% CI .90-2.81)**. We found a weaker association, non-significant as well, in the opposite direction between income and the probability of including vaccination objectives in action plans; here an increase in income is accompanied by a slightly **lower** probability **(OR = .89; p = .61; 95% CI .57-1.39)**. The multilevel model shows that regional variation is larger when it comes to the inclusion of specific vaccines in national action plans compared to objectives on vaccination (see Supplementary File 2).’

The Results section of the Abstract:

‘We found indications that vaccination objectives are more often included in AMR plans from **lower** income countries, while **higher** income countries more often include specific vaccines.’

The Discussion section:

‘Similarly, our review found indications of a possible association between the income level and a focus on objectives on vaccination, with HICs **less** likely to include vaccination objectives in their action plans.’

The original article has since been updated to correct these errors. The authors thank you for reading this erratum and apologize for any inconvenience caused.

### Electronic supplementary material

Below is the link to the electronic supplementary material.

Additional File 2

