# Supplementary material for: Assessment of the inclusion of vaccination as an intervention to reduce antimicrobial resistance in AMR national action plans: a global review
Source: Global Health. 2022 Oct 17;18:85. doi: 10.1186/s12992-022-00878-6 (PMC9574789; doi:10.1186/s12992-022-00878-6)
Supplement: Supplementary file 1 [file 12992_2022_878_MOESM2_ESM.docx]

**Supplementary File 2**

Shown here are the results of two logistic multi-level regression analyses; two levels: data from 77 countries, nested in 6 regions. The first analysis tests the effect of country income on vaccination objectives being included in AMR national action plans. The second analysis is similar but with a different dependent variable: specific vaccines are included in national action plans or not. The effect of income on the dependent variables is not significant in the two models. However, the direction of the associations is different. The association between income and vaccination objectives includes in national action plans is positive. The association between income and specific vaccines included in AMR national action plans is negative.

***Analysis 1. Vaccination objective included in national action plan predicted by income***

Mixed-effects logistic regression               Number of obs     =         77

Group variable:  Regional_gro~s                 Number of groups  =          6

                                                Obs per group:

                                                              min =          8

                                                              avg =       12.8

                                                              max =         16

Integration method: mvaghermite                 Integration pts.  =          7

                                                Wald chi2(1)      =       0.11

Log likelihood = -52.527887                     Prob > chi2       =     0.7378

---------------------------------------------------------------------------------------

Vaccination_Objective |      Coef.   Std. Err.      z    P>|z|     [95% Conf. Interval]

----------------------+----------------------------------------------------------------

         Income_group |   .0522987   .1562426     0.33   0.738    -.2539311    .3585285

                _cons |   .1658409   .4297875     0.39   0.700    -.6765272    1.008209

----------------------+----------------------------------------------------------------

Regional_groups       |

            var(_cons)|   2.13e-35   3.02e-18                             .           .

---------------------------------------------------------------------------------------

LR test vs. logistic model: chi2(0) = 0.00                Prob > chi2 =      .

Vaccination_Objective | Odds Ratio   Std. Err.      z    P>|z|     [95% Conf. Interval]

----------------------+----------------------------------------------------------------

         Income_group |    1.05369   .1646313     0.33   0.738     .7757453    1.431222

***Analysis 2. Specific vaccines included in national action plan predicted by income***

Mixed-effects logistic regression               Number of obs     =         77

Group variable:  Regional_gro~s                 Number of groups  =          6

                                                Obs per group:

                                                              min =          8

                                                              avg =       12.8

                                                              max =         16

Integration method: mvaghermite                 Integration pts.  =          7

                                                Wald chi2(1)      =       1.79

Log likelihood = -39.705251                     Prob > chi2       =     0.1815

--------------------------------------------------------------------------------------

Vaccination_Specific |      Coef.   Std. Err.      z    P>|z|     [95% Conf. Interval]

---------------------+----------------------------------------------------------------

        Income_group |  -.2651404   .1984504    -1.34   0.182    -.6540961    .1238153

               _cons |   1.923385   .5959789     3.23   0.001      .755288    3.091482

---------------------+----------------------------------------------------------------

Regional_groups      |

           var(_cons)|   7.31e-33   9.98e-17                             .           .

--------------------------------------------------------------------------------------

LR test vs. logistic model: chi2(0) = 0.00                Prob > chi2 =      .

Vaccination_Specific | Odds Ratio   Std. Err.      z    P>|z|     [95% Conf. Interval]

---------------------+----------------------------------------------------------------

        Income_group |   .7670993   .1522312    -1.34   0.182     .5199124    1.131808
